# Prediction of Relative Physical Activity Intensity Using Multimodal Sensing of Physiological Data

**DOI:** 10.3390/s19204509

**Published:** 2019-10-17

**Authors:** Alok Kumar Chowdhury, Dian Tjondronegoro, Vinod Chandran, Jinglan Zhang, Stewart G. Trost

**Affiliations:** 1 Science and Engineering Faculty, Queensland University of Technology, Brisbane 4000, Australia; alokchy04@yahoo.com (A.K.C.); vinod.chandran2@bigpond.com (V.C.); jinglan.zhang@qut.edu.au (J.Z.); 2 Department of Business Strategy and Innovation, Griffith University, Nathan 4111, Australia; d.tjondronegoro@griffith.edu.au; 3 Institute of Health and Biomedical Innovation at QLD Centre for Children’s Health Research, School of Exercise and Nutrition Sciences, Queensland University of Technology, South Brisbane 4101, Australia

**Keywords:** motion sensors, machine learning, classification, random forest, support vector machine, neural networks, relative physical activity intensity, Borg’s RPE

## Abstract

This study examined the feasibility of a non-laboratory approach that uses machine learning on multimodal sensor data to predict relative physical activity (PA) intensity. A total of 22 participants completed up to 7 PA sessions, where each session comprised 5 trials (sitting and standing, comfortable walk, brisk walk, jogging, running). Participants wore a wrist-strapped sensor that recorded heart-rate (HR), electrodermal activity (Eda) and skin temperature (Temp). After each trial, participants provided ratings of perceived exertion (RPE). Three classifiers, including random forest (RF), neural network (NN) and support vector machine (SVM), were applied independently on each feature set to predict relative PA intensity as low (RPE ≤ 11), moderate (RPE 12–14), or high (RPE ≥ 15). Then, both feature fusion and decision fusion of all combinations of sensor modalities were carried out to investigate the best combination. Among the single modality feature sets, HR provided the best performance. The combination of modalities using feature fusion provided a small improvement in performance. Decision fusion did not improve performance over HR features alone. A machine learning approach using features from HR provided acceptable predictions of relative PA intensity. Adding features from other sensing modalities did not significantly improve performance.

## 1. Introduction

Regular participation in physical activity (PA) is recognized as one of the most important steps that people can take to improve their health [1]. Physical inactivity significantly increases the risk of numerous chronic health conditions, including cardiovascular disease, type-2 diabetes, cancers of the breast and colon, and depression [2,3,4]. This extensive scientific evidence for the health benefits of PA has prompted numerous medical and public health organizations to issue recommendations or guidelines for participation in PA. For example, the World Health Organization recommends at least 150 min of moderate-intensity PA, or at least 75 min of vigorous intensity PA per week, accumulated in bouts of at least 10 min in duration [5].

In recent years, due to the increasing use of wearable sensor technology, accelerometer and heart-rate based objective PA monitoring has become popular among researchers and consumers [6,7,8]. In contrast to self-report methods, sensor-based approaches can be used to collect real-time responses from users efficiently and unobtrusively, and can track the frequency, intensity and duration of PA [9,10,11,12,13]. Such features enable users to record, view and share PA status with their health practitioners and peers. Because current PA guidelines call for participation in moderate- and vigorous-intensity PA, it is important that wearable sensor systems for monitoring PA behaviour provide accurate determinations of PA intensity.

PA intensity can be defined in relative or absolute terms. Relative intensity is generally expressed as a percentage of an individual’s maximal aerobic capacity (% VO_2_max, % VO_2_ reserve, % HR reserve) or based on ratings of perceived exertion (RPE) [14,15]. In relative terms, moderate intensity PA is typically defined as 40% to 60% of VO_2_ reserve or an RPE of 12–14 [16,17,18]. Absolute intensity, on the other hand, refers to the energy expenditure associated with a specific activity expressed in kilocalories per unit of time or as multiples of resting metabolism or Metabolic Equivalents (METs), where 1 MET is assumed to be 3.5 mL·kg^−1^·min^−1^ or approximately 1 kcal^.^kg^−1.^h^−1^. In absolute intensity terms, moderate PA is defined as an energy expenditure of 3–6 METs, regardless of an individual’s aerobic capacity. Thus, in order to achieve moderate intensity PA based on absolute intensity, individuals with a lower aerobic capacity are required to work at a significantly higher relative intensity [14,19]. For example, “brisk” walking on level ground has an absolute intensity or energy expenditure of 4 METs. For a young healthy person with a maximal aerobic capacity of 10 METs, the relative intensity is 40% of maximal capacity; whereas for an individual with a chronic health condition with a maximal aerobic capacity of 6 METs, the relative intensity is 67%. Thus, despite expending the same amount of energy during brisk walking, the two individuals are working at very different levels of relative intensity, resulting in very different physiological stimuli and training adaptations. Conversely, in relative intensity terms, less fit individuals exercising at an absolute intensity of 2.5 METs may be judged as participating in moderate intensity PA, if the work rate exceeds 40% of maximum oxygen uptake reserve.

To date, research efforts to quantify PA intensity from wearable sensors have predominantly been based on absolute intensity [9,14,19,20]. Because such estimates do not consider an individual’s aerobic fitness, age or health status, the intensity of PA could be above accepted relative intensity thresholds for moderate-to-vigorous PA (MVPA), but below the established 3 MET absolute intensity threshold for MVPA [21,22]. As such, a significant proportion of individuals with limited aerobic capacity are erroneously misclassified as not meeting PA guidelines. Moreover, m-Health platforms using wearable sensors systems to monitor the absolute intensity of PA could be encouraging individuals to exercise at relative intensities that are neither safe nor effective [23]. Thus, the development of validated algorithms to predict relative PA intensity from wearable sensor data constitutes an important research priority.

Because of the linear relationship between HR and work rate during steady state exercise, HR based indices such as percentage of maximal HR (%HRmax) and percentage of HR reserve (%HRR) are widely used metrics for quantifying the relative intensity of PA [14,24,25]. However, there are a number of drawbacks to using these metrics. First, the measurement error associated with commonly used age-based prediction equations for HRmax (220 − age, 208 − (0.7 × age)) range from 10 to 12 bpm, and therefore do not provide accurate predictions of individual HRmax [26,27]. Importantly, the measurement error for HRmax increases to ±40 bpm in patients with coronary heart disease taking beta blockers [28]. Second, patients with cardiovascular disease frequently present with chronotropic incompetence or an inability of the heart to increase its rate commensurate with increases in physical activity or metabolic demand [29]. For these patients, conventional HR based indices can significantly underestimate the true relative intensity of exercise and put patients with low exercise tolerance at risk. Third, to account for inter-individual differences in aerobic capacity and HRmax, it is necessary to personalize the relationship between HR and work rate via individual calibration in the laboratory, which is time intensive, requires expensive instrumentation, and is not feasible in large field-based studies [25,30].

Along with HR, other physiological sensor modalities including electrodermal activity (Eda) (variation of the electrical properties of the skin in response to sweat secretion) and skin temperature (Temp) can be easily measured via wearable sensors. These physiological indicators can provide valuable information about the metabolic demand of exercise, and can also be used to predict relative PA intensity [31]. However, to our knowledge, the use of multiple modalities of physiological data for relative intensity prediction has not been previously investigated, except our previous proof-of-concept work [31].

An alternative approach to measuring relative intensity that does not require instrumentation or individual calibration in the laboratory is the use of effort perception or ratings of perceived exertion (RPE). Effort perception scales such as the Borg alpha-numeric RPE Category Scale are commonly used in exercise testing and prescription contexts and have been shown to a valid and reliable indicator of relative PA intensity [17,32,33,34]. Yet, despite the widespread use of RPE for effort estimation, the utility of machine learning algorithms to classify relative PA intensity category using RPE as ground truth, has received very little research attention. If features in the signals from multiple physiological sensors can be trained to predict relative PA intensity category based on RPE, then PA intensity predictions can be more personalized, whereby users can track their PA sessions to exercise at an intensity that is both safe and effective. We have previously shown that regression models employing features in heart rate data, electrodermal activity and body temperature can predict relative PA intensity [31]. However, the RMSE of approximately two points on the Borg RPE scale suggests that such predictions may lack the precision required for real-world health monitoring applications. As such, relative intensity ratings based on categories of RPE that are easily understood by patients and end-users (i.e., low, moderate and high) may have more validity, and hence clinical utility.

To explore this hypothesis, the current study investigated the feasibility of a non-laboratory approach that uses machine learning on features in multimodal sensor data to effectively *classify* relative PA intensity categories. A non-laboratory dataset, collected from 22 people was utilized, where relative intensity categories based on Borg’s RPE scale was used as a ground truth measure of relative intensity. The features extracted from multimodal physiological data were applied to three state-of-the-art machine learning algorithms (including support vector machine, random forest and neural network) to predict three classes of relative PA intensity. The study (1) identified the best set of features from a single sensor modality for predicting relative PA intensity category, (2) explored both feature fusion and decision fusion to combine different sensor modalities to improve relative PA intensity prediction performance, and (3) identified the best combination of modality features for predicting relative PA intensity category.

## 2. Materials and Methods

### 2.1. Participants

Twenty-two adults (mean age = 29.8 ± 3.2 years; BMI = 25.3 ± 2.6; male = 77.3%) participated in the study. The inclusion criteria were: (1) age between 18 to 40 years; (2) apparently healthy and able to complete the PA protocol as measured by the Physical Activity Readiness Questionnaire (PAR-Q+); and (3) no hospitalisations within the last six months. Participants were recruited from the greater Brisbane metropolitan area via university email lists and word of mouth. All participants were confirmed to be not taking medications for any condition. Prior to participating in the study, each participant provided written informed consent. The data collection protocol was approved by the Office of Research Ethics and Integrity of the Queensland University of Technology (Ethics number: 1500000962).

### 2.2. Protocol

Participants completed up to seven weekly PA testing sessions (91% of all participants participated 5+ sessions). Each session comprised five PA trials ranging from sedentary to vigorous intensity. The activity trials were quiet sitting or standing (5 min), comfortable walk (5 min), brisk walk (5 min), jogging (3 min), and fast running (2 min). The intensity of walking, jogging and fast running trials was self-selected. Sufficient recovery time was provided between each activity trial. The resting times after each activity trial were 5 min, 15 min, 15 min, 17 min, and 18 min respectively. The resting times were sufficient to ensure that HR returned to resting level before the beginning of the next trial and were based on the domain expertise of the senior author who is an exercise physiologist. Thus, each session lasted approximately 100 min. To ensure a common outdoor environment, all sessions were performed in the same park in the afternoon. During the trials, a researcher accompanied the participants and provided verbal feedback (if required) to assist participants with motivation and to ensure an even pacing during each trial. Fifteen participants completed seven testing sessions, three participants completed six testing sessions, two participants completed five testing sessions, one participant completed two testing sessions and one participant completed one testing session. The main reasons for not completing all seven testing sessions was time conflicts with the scheduled testing session, work related travel, and illness. Of the 770 PA trials possible (22 × 7 × 5 = 770), 109 PA trials had missing or incomplete data due to participants opting not to complete the trial or terminating the trial early, leaving a total of 661 PA trials for training and testing. Of this number, 21 PA trials had missing or incomplete sensor data, with a further 25 PA trials excluded due to non-valid RPE data (i.e., reporting an RPE of less than 10 for each trial), leaving a total of 615 PA trials for analysis.

### 2.3. Data Acquisition

Before the first testing session, participants provided basic profile information such as age, sex, height, and weight. Habitual PA level was measured using the Active Australian Survey [35]. Responses to the survey were used to estimate PA status (sedentary, insufficiently active, or sufficiently active) based on reported total activity time and total number of activity sessions.

During each session, participants wore an Empatica E4 wrist-watch (Boston, MA, USA), a Polar H7 chest strap heart-rate monitor, and a mobile phone to record the HR data. The Empatica E4 was worn on the non-dominant wrist, and captured electrodermal activity (Eda) and skin temperature (Temp) [36]. The sampling rate for Eda and Temp data were 4 Hz. The Polar heart-rate monitor recorded heart-rate (HR) in beats-per-minute at a sampling rate of 1 Hz and RR-interval data.

### 2.4. Ground Truth Annotation

Relative intensity was measured using the Borg Rating of Perceived Exertion (RPE) scale [32,33,37]. The scale was presented and explained to the participants before performing each session. Immediately after each trial, participant rated their perceived exertion for the entire PA trial by pointing to the printed Borg’s RPE scale. Over the 7-week study period, RPE ratings for each activity trial exhibited a high degree of repeatability with a single measure ICC of 0.92 (95% CI = 0.89–0.95). Borg RPE ratings were categorised into three relative intensity classes corresponding to low (6–11), moderate (12–14) and high intensity (15–20) [18].

### 2.5. Relative Intensity Category Prediction System

A relative PA intensity category prediction system was designed by applying machine learning algorithms to predict low, moderate and high relative intensity from features in the raw sensor data. The overall framework of the system consisted of five steps: pre-processing, feature extraction, normalisation and feature selection, classification, fusion and evaluation.

#### 2.5.1. Pre-Processing

HR, Eda and Temp data were annotated with the relative intensity classes and transformed into time-series data structure. Only the data collected during the activity trials underwent annotation. To remove motion artefacts in the physiological data, a moving average filter with a span of 5 s was applied. In addition, data from the first and last 10 s of each trial was discarded to ensure that the participants were at a steady pace and not building up speed at the beginning or slowing down toward the end of each trial.

#### 2.5.2. Feature Extraction

A number of time and frequency domain features were extracted from each sensor modality. Because participants reported RPE at the end of each activity trial, the window size for feature extraction was set equal to the duration of the activity trial. For purposes of comparison, we trained models on features extracted from the last 60 s of each activity trial. The results of these analyses are reported in the supplementary content. The extracted features from the sensor modalities are given in Table 1.

Each of these feature sets were combined with *participant attribute data or person level features*. Person level features included height, weight, age, BMI, gender and PA status.

#### 2.5.3. Normalisation and Feature Selection

In order to limit features to a common range and optimize the quality of input data, linear methods were used to normalise each feature to a zero mean and unit variance. Because some features can be redundant and provide irrelevant information which can undesirably affect performance, a minimum-redundancy–maximum-relevance feature selection (MRMR) method was applied [38] on the complete dataset. As minimum redundancy criteria, this method used minimum mutual information between features; and for maximum relevance criteria, it used the maximal mutual information between the classes and feature. This approach resulted in the selection of only the best 15 features as inputs to the classifiers.

### 2.6. Classification Algorithms

Three state-of-the-art machine learning algorithms, which are prominently used in PA domain, including support vector machine (SVM), random forest (RF), and neural network (NN) were utilised. In our implementation, two-class SVM was adapted in a fashion that firstly classified one class against all other classes and then classified another class versus the remaining classes and so on [11]. A radial basis function kernel function was chosen for use in the SVM classifier. RF was implemented using the “Treebagger” classification tool within Matlab (2017a, The MathWorks Inc., Natick, MA, USA). The number of decision trees in each RF classifier was empirically set to 100 because it provided optimum performance compared to 50 and 150. For the NN, the number of input, hidden and output neurons were 10, 7, and 3 respectively. The maximum epoch and learning rate were set to 250 and 0.00, respectively [39]. The Matlab code and data are available on request.

### 2.7. Fusion to Combine Multiple Sensor Modalities

In order to find the best feature combination for relative intensity prediction, both feature-level and decision-level fusion were performed. In feature-level fusion, all combinations of the three sensor feature sets were merged together. Then, feature selection and classification algorithms were applied on the merged feature set. In the decision-level fusion, each of the sensor feature sets made an independent decision, which were then combined using our previously reported posterior-adapted class-based decision fusion algorithm [10]. This algorithm first trains a single classifier for each sensor modality to gain an understanding of each model’s relative prediction performance across classes. Then, for each single modality classifier it assigns weights to the classes (class-based weights) based on performance in the training data. During testing of new data, each classifier provides a decision with posterior probability which is subsequently used to adjust the class-based weights (posterior-adapted class-based weights). In the fusion step, the class with the highest posterior-adapted class-based weight is selected as the final predicted class.

### 2.8. Performance Evaluation

Performance was evaluated using leave-one-subject-out (LOSO) cross-validation [11,40]. In LOSO, data from one user are used for testing, the other users samples are used for training. In this way, samples of each subject are used exactly once for testing. This study used F1 score [41] to measure the performance of each classifier. The study favoured F1 score over classification accuracy because unlike accuracy or percentage of agreement, it is not influenced by class distribution. The F1 score was computed from precision and recall by keeping a balance between them.

(1) $$F1\;Score=\;\frac{2*\;precision*\;recall}{precision+recall}*\;100\%$$

Precision describes the exactness of a classifier. A lower value of precision indicates a high false-positive rate.

(2) $$precision=\;\frac{true\;positives}{true\;positives+false\;positives}*\;100\%$$

Recall or sensitivity is useful to measure the completeness of classifiers. Low recall indicates a high false-negative rate.

(3) $$recall=\;\frac{true\;positives}{true\;positives+false\;negative}*\;100\%$$

### 2.9. Statistical Comparison

Performance differences for the three sensor modalities and their combinations based on feature fusion were tested for statistical significance using one-way repeated measures ANOVA. The F1-scores for all folds/users for all classifiers were merged to increase the statistical power and enhance the generalizability of the findings. We elected not to test the differences between the models based on decision fusion because all classifiers based on the combination of sensors failed to surpass the performance of the single modality models.

## 3. Results

### 3.1. Relative Intensity Classification from a Single Modality

Table 2 reports F1 scores for classifiers trained on features from a single modality. A similar pattern of results was observed across the three supervised learning algorithms. HR features provided the highest classification accuracy, with SVM achieving the highest accuracy (84.6%). Performance was consistently low for the classifiers trained on the Eda or Temp feature sets.

### 3.2. Feature Fusion Results

**Figure 1:**
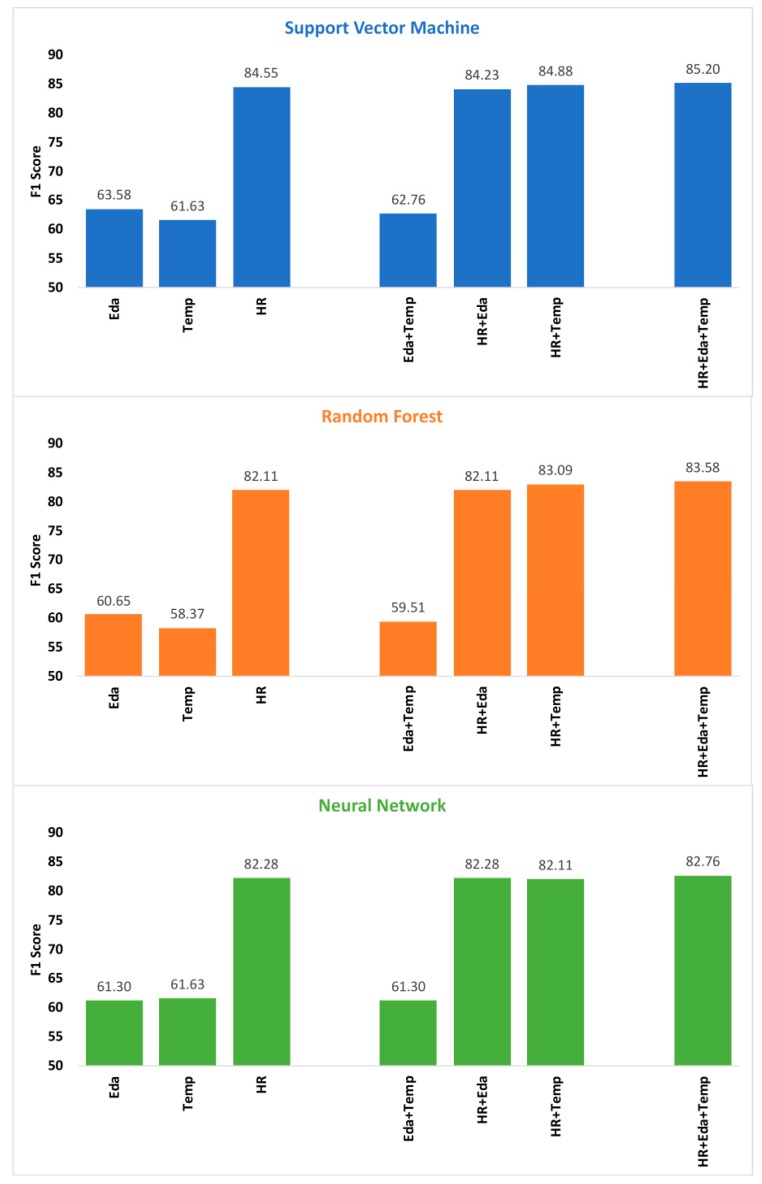
F1 Scores for all combinations of modalities using feature fusion; Note: results of single modalities are also given as base-lines. Abbreviations: Eda = electrodermal activity; HR = heart-rate; Temp = temperature.

Figure 1 reports feature fusion results (F1 scores) for all possible combinations of the three sensor modalities using three supervised learning algorithms. The features selected in these models are reported in the supplementary content. For all three classifiers, the combination of HR, Eda and Temp (HR + Eda + Temp) provided the best performance, but the margin of improvement over HR alone was small. Compared to HR only, the combination of features from HR + Eda + Temp yielded performance improvements of 0.7%, 1.5% and 0.5% for SVM, RF, and NN, respectively. The combination of HR and temperature (HR + Temp) marginally improved the performance of the SVM and RF classifiers; however, for all classifiers, the combination of HR and Eda failed to exceed the performance of HR alone.

**Figure 2:**
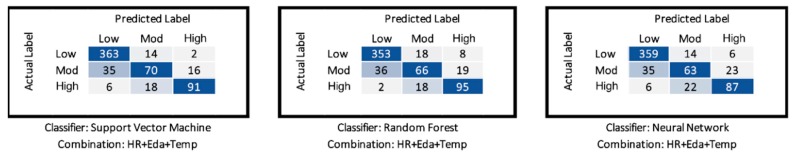
The confusion matrices for the best combinations in each classifier using feature fusion. Abbreviations: Eda = electrodermal activity; HR = heart-rate; Mod = moderate; Temp = temperature.

The confusion matrices for the best performing combination (HR + Eda + Temp) are shown in Figure 2. In all cases, classifiers correctly classified a very high percentage of the low (93–96%) and high (76–83%) relative intensity activity trials and a majority of the activity trials with moderate relative intensity (52–58%). However, for the moderate intensity trials, 29–30% was misclassified as low intensity and 13–19% as high intensity.

### 3.3. Decision Fusion Results

**Figure 3:**
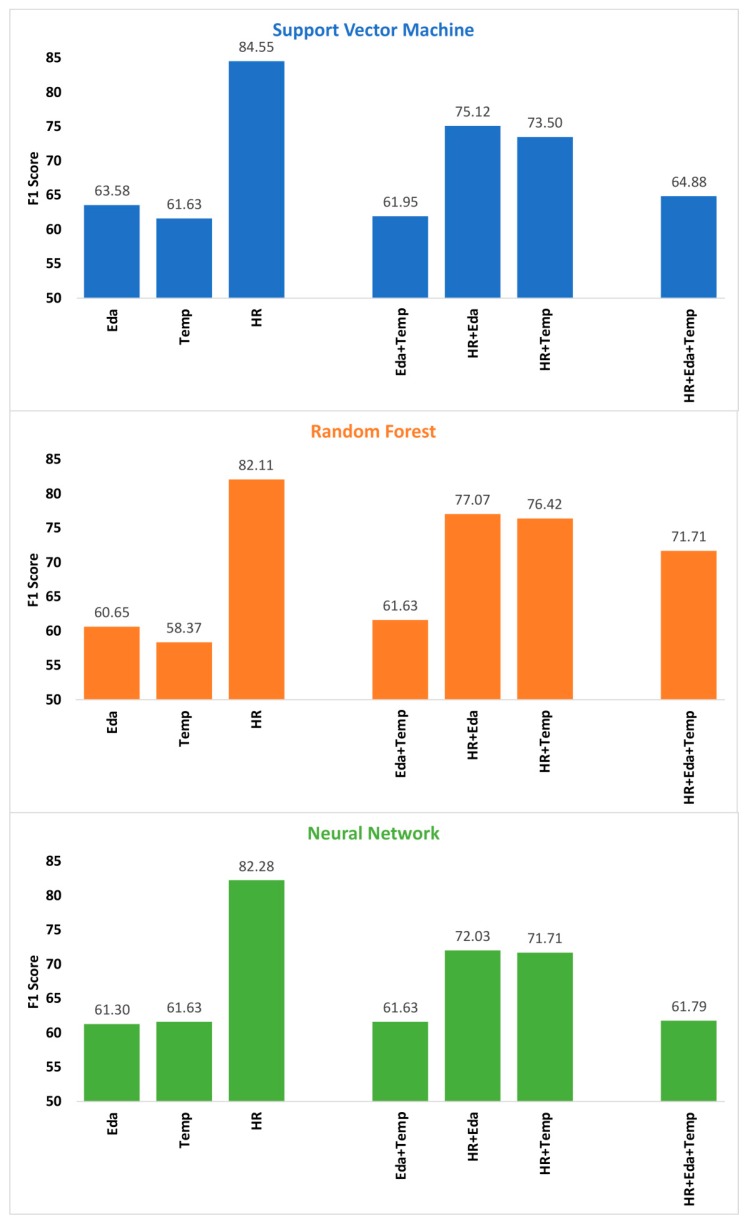
Scores for all combinations of modalities using decision fusion; Note: results of single modalities are also given as base-lines. Abbreviations: Eda = electrodermal activity; HR = heart-rate; Temp = temperature.

Figure 3 shows the decision fusion results (F1 scores) of all combinations of modalities using three supervised learning algorithms. None of the combinations based on decision fusion exceeded the performance of the single modality HR classifiers. In fact, the addition of Eda and Temp to the HR classifiers reduced the performance.

### 3.4. Statistical Comparison Results

Overall, mean F1-scores for the three sensor modalities and their combinations based on feature fusion differed significantly (Wilks’ Lambda = 0.247, F (6, 57) = 29.002, *p* < 0.0001). Fisher LSD post hoc comparisons revealed that classifiers trained on HR features provided significantly better performance than those trained on Eda or Temp features (*p* < 0.05). None of the combination modalities provided significantly improved performance relative to the HR only modality.

## 4. Discussion

This study systematically investigated the use of machine learning algorithms trained on features in multimodal physiological data to classify relative PA intensity category. Across all supervised learning algorithms (SVM, RF and NN), classifiers trained on time and frequency domain features extracted from heart-rate data provided the best performance compared to those trained on features extracted in electrodermal activity and skin temperature. Two fusion techniques (feature- and decision-fusion) were examined to identify the effective combination of the physiological data. Of the two, feature fusion marginally improved performance when adding electrodermal activity and skin temperature features to the heart-rate feature set, with SVM providing the best performance (85.2%). None of the combinations of modalities provided statistically significant improvements in performance relative to the single heart-rate modality classifiers.

Most previous studies have used HR indices such as beats per minute, mean HR, and maximal HR to monitor relative intensity and few have utilised RR interval data to classify relative PA intensity [14,24,25]. In the current study, time and frequency- domain features extracted from RR interval data and simple time-domain features extracted from HR data were combined and used as a set of HR features. RR interval represents the beat-to-beat fluctuations of the heart-rate, and the magnitude and underlying frequency of the decrease in RR interval that occurs with progressive exercise may be a more informative indicator of relative PA intensity than simple changes in HR [42]. Electrodermal activity and skin temperature have also been linked to relative PA intensity [43]. However, the classifiers based on these modalities alone did not provide satisfactory classification accuracy. Electrodermal activity or galvanic skin response is strongly related to sympathetic activation and maybe a more reliable indicator of stress or emotional arousal than relative PA intensity [44], particularly during mild exercise where increases in HR are achieved by the withdrawal of vagal tone [45,46]. Further, skin temperature during exercise is affected by a multitude of factors, including mode of exercise, thermoregulatory capacity, body composition and ambient temperature [47], and may be a less reliable indicator of physiological strain or relative PA intensity.

When features from the different sensor modalities were combined with feature fusion, classification performance improved, but only marginally. This indicates that features from these modalities provide relatively little additional information to that provided by HR features. When the decisions of the single modality classifiers were combined using decision fusion, the combinations showed considerably inferior performance. In decision fusion, classifiers from each sensor modality made independent predictions of the relative intensity and then a posterior-adapted class-based weighted algorithm combined those decisions. In our earlier research, posterior-adapted class-based algorithm showed improved performance for PA recognition when combining the accelerometer data obtained from multiple body locations (ankle, chest, and wrist) [10]. In that study, PA recognition at each location had comparable but complementary performance accuracy. Thus, individual decisions from each model performed better when combined using posterior-adapted class-based weighted algorithm. In the current study, decision fusion showed poor performance because electrodermal activity and skin temperature did not provide satisfactory performance in their own right. Thus, combining the decision of two relatively poorly performing prediction models led to further decrements in overall performance.

In the confusion matrices, it can be seen that most of the low and high relative intensity trials were classified correctly. Very few high intensity trials were misclassified as low intensity which is important in exercise monitoring applications serving low-fit individuals with chronic health conditions among whom high intensity exercise may be unsafe. Conversely, low intensity exercise trials were infrequently misclassified as moderate or high intensity, which is important for end users wishing to exercise at a relative intensity that is effective for promoting health and cardiorespiratory fitness. However, for moderate relative intensity, classification accuracy was modest. It may be that differentiating moderate intensity exercise from low- or high-intensity using the Borg RPE scale is more challenging for individuals, and that the relationship between features in the physiological signals and perceived effort during moderate intensity exercise is more complex. From an application point of view, it may be less problematic to misclassify moderate intensity as vigorous; (and vice versa) as both are counted toward meeting PA guidelines. But, the misclassification of moderate intensity PA as low intensity is problematic. Therefore, further research is needed to improve the model’s performance or reduce misclassification between low and moderate intensity, such as personalising the physiological data before inputting into the classifier.

A strength of the current study was the use of machine learning on the multimodal physiological data, collected in a non-laboratory environment, to predict the relative intensity category. The examination of each of these modalities independently or combined (using fusion), were among the main strengths. The use of three different “state-of-the-art” classification algorithms was an additional strength. Furthermore, the repeatability of RPE ratings for each exercise trial was carefully monitored over multiple sessions and shown to be very consistent, reinforcing its use as a valid option for ground-truth assessment of relative PA intensity. There were, however, some limitations that warrant consideration. First, although activity trials were self-paced and completed at a user-specified intensity, the data were collected in predetermined sequences with known duration. Thus, additional work is required to evaluate the performance of the proposed methods in true free-living contexts. Second, only ambulatory activities were included in training data. Future studies should include a more diverse set of lifestyles activities ranging in relative intensity from sedentary to vigorous. Third, the window size for feature selection was set to match the duration of the activity trial. In future work, we will explore the optimal window size required for accurate classification of relative exercise intensity in real-world monitoring scenarios. We anticipate that our algorithms will be used to monitor and classify relative exercise intensity in real time in clinical populations with low exercise tolerance, for whom such monitoring is indicated. End users would be able to select an exercise monitoring or training mode at the beginning of a planned exercise session and de-select the mode when the exercise session is completed. We also plan to explore other data segmentation approaches, including the implementation of an activity recognition algorithm to flag bouts of purposeful exercise. Fourth, although some previous works found personalising physiological data can reduce inter-individual differences and improve the accuracy of EE or PA recognition [48], our experiment was unable to personalise the physiological features due to absence of resting data or sleep HR. Fifth, our study developed and tested models using data from healthy young adults. Accordingly, future studies should test our approach in age diverse samples with low exercise tolerance. Fifth and finally, our study only focused on the use of multimodal physiological data for relative intensity prediction. In the future, a study can be carried out to investigate the utility of the combination of biomechanical and physiological data for relative intensity prediction.

In summary, the results demonstrate that relative PA intensity classification can be performed by using machine learning on multimodal physiological data. Of the different modes of physiological data examined, features extracted from HR data provided the best performance. Although classifiers trained on non-heart rate features such as electrodermal activity and skin temperature cannot provide satisfactory results on their own, they can marginally improve the classification accuracy when combined with HR features. Thus, the best single sensor modality out of the three included in the present study was heart-rate.

## Figures and Tables

**Table 1:** Feature set extracted from each sensor modality.

**1. HR Feature Set**	*The features from both HR in beats-per-minutes and RR interval data were extracted and used as a HR feature set.* **Features extracted from HR in beats-per-minutes** *Time domain features:* mean, variance, standard deviation, skewness, kurtosis, median, numerical gradient, on and off response, the number of times HR increased normalised for window size, and the number of times HR decreased normalised for window size. **Features extracted from RR interval** *Time domain features:* mean, variance, standard deviation, skewness, kurtosis, median, standard deviation of successive differences between adjoining normal cycles (SDSD), Square root of the mean squared difference of successive RR-intervals (rMSSD), Number of pairs of successive RR-intervals that differ by more than 20 ms/length (pNN20), Number of pairs of successive RR-intervals that differ by more than 50 ms/length (pNN50). *Frequency features:* spectral energy density (aVLF, aLF, aHF), relative power (pVLF, pLF, pHF), and normalised power (nLF, nHF) of very low frequency (0–0.04 Hz), low frequency (0.04–0.15 Hz), and high frequency (0.15–0.40 Hz) components, total spectral energy density (aTotal), and ratio between LF and HF band energy (LF/HF).
**2. Eda Feature Set**	*Time domain features:* mean, variance, standard deviation, skewness, kurtosis, and median
**3. Skin Temp Feature Set**	*Time domain features:* mean, variance, standard deviation, skewness, kurtosis, and median

Abbreviations: Eda = electrodermal activity; HR = heart-rate; RR = R to R interval; Temp = temperature.

**Table 2:** F1-scores of classifiers trained on single modality feature sets.

Feature(s)	SVM (F1 Score %)	RF (F1 Score %)	NN (F1 Score %)
Eda	63.58	60.65	61.30
Temp	61.63	58.37	61.63
HR	84.55	82.11	82.28

Abbreviations: Eda = electrodermal activity; HR = heart-rate; NN = neural network; RF = random forest; SVM = support vector machine; Temp = temperature.

## Supplementary Materials

The following are available online at https://www.mdpi.com/1424-8220/19/20/4509/s1.
