# Supplementary material for: Prediction of Relative Physical Activity Intensity Using Multimodal Sensing of Physiological Data
Source: Sensors (Basel). 2019 Oct 17;19(20):4509. doi: 10.3390/s19204509 (PMC6833090; doi:10.3390/s19204509)
Supplement: Supplementary file 1 [file sensors-19-04509-s001.pdf]

Supplementary Content

To explore the impact of window size of model performance we trained the classification models using data from the last 60 s of each activity trial. Although the findings relative to the contribution of each sensor modality and their fusion were qualitatively similar, F1 scores dropped significantly when using the last 60 s of the activity trial – but only for HR features. This suggested that EDA and Temp signals were more stable across the duration of the activity than HR. Depending on the task, HR increased over the duration of the trial, which may explain why features in the HR data were the best predictors of relative exercise intensity.

Table S1. Feature Fusion (last 60 sof each activity trial).

Feature(s)	SVM (F1 Score %)	RF (F1 Score %)	NN (F1 Score %)
Eda	62.70	57.33	60.75
Temp	61.89	59.45	42.35
HR	75.57	75.73	71.17
Eda+Temp	62.70	62.87	63.03
HR+Eda	76.55	77.69	71.34
HR+Temp	76.71	77.85	76.22
HR+Eda+Temp	77.36	78.50	72.80

Table S2: Decision Fusion (last 60 s of each activity trial).

Feature(s)	SVM (F1 Score %)	RF (F1 Score %)	NN (F1 Score %)
Eda	62.70	57.33	60.75
Temp	61.89	59.45	42.35
HR	75.57	75.73	71.17
Eda+Temp	61.89	62.22	61.40
HR+Eda	65.64	72.96	63.36
HR+Temp	63.03	70.52	66.61
HR+Eda+Temp	63.84	68.89	63.68
